# Implementation of a routine outcome monitoring and feedback system for psychotherapy in Argentina: A pilot study

**DOI:** 10.3389/fpsyg.2022.1029164

**Published:** 2023-01-05

**Authors:** Juan Martín Gómez-Penedo, Rocio Manubens, Malenka Areas, Javier Fernández-Álvarez, Manuel Meglio, Anna Babl, Santiago Juan, Agnese Ronchi, Roberto Muiños, Andrés Roussos, Wolfgang Lutz, Martin grosse Holtforth

**Affiliations:** ^1^Equipo de Investigación en Psicología Clínica, Laboratorio de Análisis Estadísticos, Secretaría de Investigaciones, Facultad de Psicología, Universidad de Buenos Aires, Buenos Aires, Argentina; ^2^Consejo Nacional de Investigaciones Científicas y Técnicas, Buenos Aires, Argentina; ^3^Departamento de Personalidad, Evaluación y Tratamientos Psicológicos, Universidad Jaume I, Castellón de la Plana, Spain; ^4^Clinical Psychology and Psychotherapy Department, Institute of Psychology, University of Bern, Bern, Switzerland; ^5^Gordon F. Derner School of Psychology, Adelphi University, Garden City, NY, United States; ^6^Clinical Psychology and Psychotherapy Department, University of Trier, Trier, Germany; ^7^Psychosomatic Competence Center, University Hospital Insel, Bern, Switzerland

**Keywords:** ROM, feedback, monitoring, psychotherapy outcome, baseline characteristics

## Abstract

**Introduction:**

Routine Outcome Monitoring (ROM) has emerged as a strong candidate to improve psychotherapy processes and outcome. However, its use and implementation are greatly understudied in Latin-America. Therefore, the aim of the present pilot study conducted in Argentina was to implement a ROM and feedback system grounded on a psychometrically sound instrument to measure session by session outcome in psychotherapy.

**Methods:**

The sample consisted of 40 patients and 13 therapists. At baseline, the patients completed the Patient Health Questionnaire-9 and the Generalized Anxiety Disorder-7, and they also completed the Hopkins Symptom Checklist-11 before each of the first five sessions. To estimate patient change during the first sessions, we conducted a quantitative analysis using Hierarchical Linear Models. Furthermore, we conducted a qualitative analysis using Consensual Qualitative Research to analyze therapist perception regarding the ROM and feedback system.

**Results:**

Results showed a significant reduction in patients’ symptomatic severity during the first five sessions. Additionally, baseline depression significantly predicted the estimated severity at the end of the fifth session. Feedback was given to the therapists after the first four sessions based on these analyses. With regard to the perception of the feedback system, clinicians underlined its usefulness and user-friendly nature. They also mentioned that there was a match between the information provided and their clinical judgment. Furthermore, they provided suggestions to enhance the system that was incorporated in a new and improved version.

**Discussion:**

Limitations and clinical implications are discussed.

## Introduction

Although there is vast evidence that supports the efficiency of several psychotherapeutic interventions, approximately 30% of patients do not improve and 10% even present reliable deterioration (Barkham and Lambert, 2021). Routine Outcome Monitoring (ROM) has emerged as a relevant clinical tool to identify those patients who do not progress over the course of treatment (Lambert and Harmon, 2018). Utilizing the data collected during treatment to give feedback to therapists has been shown to significantly contribute to the improvement of psychotherapy. This additional and continuous information may prevent treatment failure, which is poorly identified by average clinicians who tend to overestimate their therapeutic performance (Lambert and Shimokawa, 2011).

The most recent and comprehensive meta-analysis, including both youth and adult populations, demonstrated a small but significant effect of feedback on symptom reduction, not on track cases and the odds of dropout (de Jong et al., 2021). Concretely, this meta-analysis including both controlled and uncontrolled primary studies showed that the use of feedback reduced dropout by 20%. These results build upon eight previous meta-analyses which also showed benefits of feedback regarding treatment duration and the odds of recovery (de Jong et al., 2021). Together, the nine existing meta-analyses form a robust body of literature supporting feedback as a meaningful potentiation of psychotherapy, given that it constitutes a simple add-on to the standard procedure.

Despite the utility of ROM and feedback, certain conditions and contexts moderate its effectiveness. There are some studies showing that in severely disturbed patients, ROM leads to better outcomes (Zilcha-Mano and Errázuriz, 2017). In the case of personality disorders, considering the cluster taxonomy, cluster B and C did not seem to benefit from ROM and feedback (de Jong et al., 2018). Moreover, as described by de Jong et al. (2021), using raw scores to provide feedback is less effective than (a) using benchmark scores to compare the score with the expected recovery trajectory or (b) automatized tools that provide instant feedback when the system detects that a patient is not on track. The literature has also identified that providing feedback to therapists and patients is more effective than only to therapists (de Jong et al., 2014).

While there is growing evidence supporting the effectiveness of ROM and feedback as a resource for a vast array of settings, clinical conditions and treatment modalities, its implementation in the real world remains a daunting task with several barriers (Boswell et al., 2015; Cooper et al., 2021; Lutz et al., 2022). These obstacles are organizational, technological, practical, attitudinal, and competency-related (Lambert and Harmon, 2018). Qualitative approaches have largely explored both therapist’s and patient’s perspectives and attitudes toward ROM. Whereas these methods have shown the promising potential of ROM and feedback for research and practice in psychotherapy, they also helped to identify the barriers that they entail (Moltu et al., 2018; Solstad et al., 2019). One main limitation of the currently available research is that almost all the evidence comes from university settings in the United States and some high-income European countries. Although this is more the norm than an exception in current scientific research, the populations analyzed using ROM and feedback are particularly defined by these characteristics, restricting generalizability to other settings and populations (Lutz et al., 2021).

Particularly in Latin America these methods remain underdeveloped, with few existing endeavors to implement and test their effectiveness (Areas et al., 2018; Paz et al., 2021; Gómez et al., 2022). The aim of the present pilot study was thus to implement a ROM and feedback system grounded on a psychometrically sound instrument to measure session by session outcome in psychotherapy in Argentina. We further aimed to understand the potential of the instrument and to identify possible obstacles in its implementation on a larger scale.

## Materials and methods

### Patients

The final sample included 40 patient-therapist dyads in naturalistic treatments of whom 34 patients completed the evaluation process. The only inclusion criterion was patient age, between 18 and 65 years old. We did not set any diagnostic exclusion criteria. The mean age was 30.2 years (SD = 7.3) ranging from 19 to 46 years. About 60% were male, and most participants had a university degree (65%). Over 77.5% of the participants were employed, the rest were students (12.5%) or unemployed (10%). About 50% were single and 50% were in a romantic relationship. 70% of the participants had previous psychotherapy treatments with an average of 2.03 (SD = 1.28) prior experiences. Mental disorders were diagnosed using the Diagnostic and Statistical Manual of Mental Disorders (DSM-V; American Psychiatric Association, 2013) by their treating therapists. Almost 50% of the patients presented an anxiety disorder, 7.5% a depressive disorder and 15% dropped out before concluding diagnosis. Descriptive information is shown in Table 1.

**Table 1:** Diagnostic characteristics of the patients (*N* = 40).

Diagnostic provided by clinician	*N*	%
Unspecified anxiety disorder	10	25
Generalized anxiety disorder	4	10
Social anxiety disorder	2	5
Panic disorder	2	5
Panic attack	1	2.5
Specific phobia	1	2.5
Unspecified depressive disorder	1	2.5
Persistent depressive disorder (dysthymia)	1	2.5
Premenstrual dysphoria disorder	1	2.5
Adjustment disorder	2	5
Post-traumatic stress disorder	1	2.5
Stress	1	2.5
Borderline personality disorder	2	5
Avoidant personality disorder	2	5
Relationship problems	2	5
Erectile disorder	1	2.5
No diagnosis	6	15
Total	40	

All diagnoses were assessed using the fifth edition of the Diagnostic and Statistical Manual of Mental Disorders (DSM-V; American Psychiatric Association, 2013); *N*, Sample number; %, Percentage.

### Therapists

The sample of therapists consisted of 13 clinicians (out of 15 that were invited), who had at least 1 year of clinical experience after clinical training (average clinical experience was 9.38 years [SD = 5.2]). Most of the therapists were male (61.5%) and the average age was 36 years (SD = 4.24). All therapists conducted on-line treatments (i.e., during the COVID-19 pandemic), with an average of 3.07 (SD = 1.32) patients per therapist, ranging from 1 to 5. As presented in Table 2, most of the clinicians self-identified as Cognitive-Behavioral therapists (53.8%).

**Table 2:** Self-identified theoretical orientation of the psychotherapists (*N* = 13).

Theoretical frameworks	*N*	%
Cognitive behavioral therapy (CBT)	7	53.8
Psychodynamic therapy	2	15.4
Evidence-based psychotherapy (EBP)	2	15.4
Humanistic-existential therapy	1	7.7
Bioenergetics	1	7.7
Total	13	

*N*, Sample number; %, Percentage.

### Materials

**Hopkins Symptom Checklist-11** (HSCL-11; Lutz et al., 2006) [Spanish Version; Gómez Penedo et al., 2021]. An 11-item self-reported scale which evaluates different psychopathological manifestations, mostly anxiety and depressive symptoms. This measure was used to assess therapeutic change and provide process feedback. Scores range from 1 (not at all) to 4 (a lot). In Argentina, this tool has shown good internal consistency (Cronbach’s α = 0.81; Gómez Penedo et al., 2021).

**Patient Health Questionnaire-9** (PHQ-9; Kroenke et al., 2001) [Spanish Version; Urtasun et al., 2019]. A 9-item self-report questionnaire that evaluates depressive symptoms. Items are based on the Diagnostic and Statistical Manual of Mental Disorders (DSM-IV) criteria. Scores range from 0 (not at all) to 3 (nearly every day) scale. In Argentina, the questionnaire showed evidence for internal consistency (Cronbach’s α = 0.87; Urtasun et al., 2019).

**Generalized Anxiety Disorder-7** (GAD-7; Spitzer et al., 2006) [Spanish Version; García-Campayo et al., 2010]. A 7-item self-report measure to assess frequency and severity of anxiety symptoms. Scores range from 0 (not at all) to 3 (nearly every day). The Spanish version has shown good internal consistency in Argentina (Cronbach’s α = 0.93; García-Campayo et al., 2010).

### Procedure

Once therapists accepted to participate in the project, they invited patients to take part in the study during their initial screening. Therapists explained the aims and terms of participation. Once patients accepted, they were sent (1) a first baseline evaluation which included sociodemographic data, the PHQ-9 and the GAD-7; (2) repeated measure evaluation which comprised the HSCL-11 for outcome monitoring. Then, for the next four sessions, patients completed the repeated measure evaluation before each session. Patients received a link by an instant messaging platform that redirected them to a website specialized in data collection for research called *SurveyMonkey*®. Data was analyzed during the period of assessment for the purpose of giving feedback to the therapists. Based on therapists’ response, changes were introduced into the feedback device (e.g., a more detailed description was added to the graphics). After the first five sessions, each therapist received an individual analysis for each patient. Finally, the research team sent questions to the therapists to receive feedback on the system and its usefulness. Both patients and therapists completed written informed consents before the start of data collect.

### Data analysis

#### Quantitative analysis

All analyses were performed in RStudio v. 1.4.1717 (RStudio Team, 2021). Specifically, we used packages psych (Revelle, 2021) and lme4 (Bates et al., 2015). Considering that we had repeated measures of HSCL-11 nested within patients, we decided to use Hierarchical Linear Models (HLMs; Raudenbush and Bryk, 2002; Gómez Penedo et al., 2019). These models account for the dependency of the data when there is a nested structure, providing more robust estimations of the parameters. Furthermore, they provide an efficient way of handling missing data mimicking an intent-to-treat approach (Westra et al., 2016).

First, we ran an unconditional model (i.e., without any predictor) to compute Intraclass Correlation Coefficients (ICC) representing the percentage of variability explained by the patient level (Raudenbush and Bryk, 2002).

Second, we ran time-as-only-predictor models to estimate the change of symptom severity over time during the first five sessions of therapy. In detail, we calculated and compared two-level linear (i.e., only with a linear term) and two-level quadratic models (i.e., with both linear and quadratic terms) including time as the only level 1 predictor (defined as sessions and centered at session five). These models allow to estimate the change in symptom severity session by session and to identify which model fits better in relation to the change trajectory.

Third, once we had selected the final time-as-only-predictor model, we ran a two-level conditional model with time as a level 1 predictor and baseline depression and anxiety as level 2 predictors of the intercept and time effects.

#### Qualitative analysis

For the qualitative analysis, we sent five questions to the therapists requesting feedback on the implemented system. The questions were: (1) *What do you think about the information provided by the feedback system?;* (2) *Do you find this information useful?;* (3) *Would you like to change anything about the system?*; (4) *Would you like to share the information from the feedback system with your patient at some point?*; (5) *Do the initial severity levels and evolution match your clinical perception of the patients’ clinical baseline level and early change?*

To analyze the collected data, a consensual qualitative analysis methodology known as Consensual Qualitative Research (CQR, Hill et al., 2005; Juan et al., 2011) was used. This method is a highly structured qualitative approach grounded on a consensus among the analysis team. This team is structured by a group of judges (“primary group”) that analyzes the data from multiple perspectives and needs to reach a consensus regarding the analysis. Then the material is sent to an auditor that checks and revises the work and has to reach a final consensus with the group. The primary group consisted of two junior researchers who had received prior training in the methodology by a senior investigator expert in CQR who was the auditor of the analysis. Based on CQR procedures, the primary group and auditor created (a) domains (thematic area that synthesizes the main topics in the analyzed material), (b) core ideas (summaries of the main notions presented in the material that should remain as close as possible to the explicit perspectives and meanings of each participant), and (c) categories (constructs that seek to group similar core ideas from different cases, involving a cross-analysis of all the participants) for classifying and analyzing the data.

## Results

### Feedback system developed

**Figure 1:**
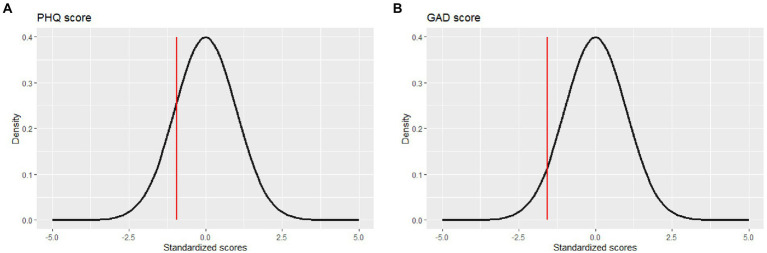
Examples of the first plots developed to provide feedback to the therapists about patients’ baseline depression **(A)** and anxiety levels **(B)**.

**Figure 2:**
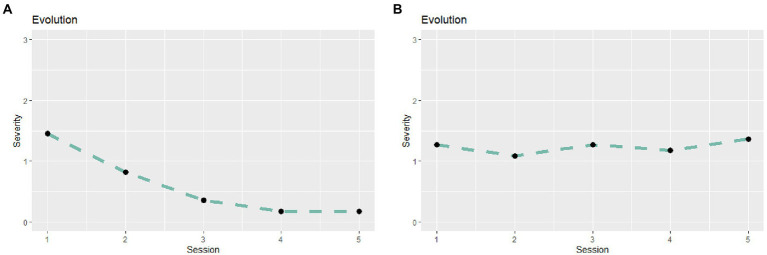
Examples of the first plots provided to the therapists to give feedback about patients’ clinical evolution during the first five sessions. **(A)** presents an example of a patient who improved during the timeframe, while **(B)** presents the example of a patient who presented a stable pattern.

The feedback was provided to the therapists based on the selected baseline variables and the multilevel analyses described above. After session five, the therapists received a first version of the feedback including baseline measures of depression and anxiety (see Figure 1) as well as repeated measures of clinical severity (see Figure 2).

Figure 1A shows an example of the graphical visualization of the PHQ-9 baseline measure. In this case, the patient showed a very low level of depression symptomatology. Figure 1B shows an example of the GAD-7 baseline measure of a patient with low levels of anxiety. The graphic included patients’ severity measure and clinical population average.

Furthermore, Figures 2A,B show examples of two trajectories of clinical severity during the first five sessions from patients of the study that were presented as feedback to the therapists. Figure 2A shows a decreasing trajectory, while Figure 2B shows a stable trajectory.

### Sample descriptives

In order to unify criteria among the different instruments used, analyses were performed based on the mean scores of each measure. Mean values in the three instruments range between a minimum value of 0 and a maximum value of 3.

Table 3 shows patients’ initial severity measures including mean and standard deviation (PHQ-9 and the GAD-7). The mean score of the total PHQ-9 was 0.95 with a SD = 0.55 (sum score Mean = 8.58 and SD = 5.11), and the mean score of the total GAD-7 was 1.40 with a SD = 0.65 (sum score Mean = 9.82 and SD = 4.57). The table also shows the descriptive analysis of patient severity scores in the first five therapy sessions. Results show a progressive decrease in patients’ severity session by session.

**Table 3:** Descriptive results of the total scores of depressive symptomatology (PHQ-9), anxious symptomatology (GAD-7) and severity levels of patients session-by-session (HSCL-11).

Measures	Session 1^*^		Session 2		Session 3		Session 4		Session 5	
	*N*	M (SD)	*N*	M (SD)	*N*	M (SD)	*N*	M (SD)	*N*	M (SD)
PHQ-9	40	0.95 (0.55)								
GAD-7	40	1.4 (0.65)								
HSCL-11	36	1.0 (0.61)	35	0.84 (0.64)	35	0.76 (0.56)	33	0.63 (0.49)	31	0.58 (0.49)

*N*, Sample number; M, Mean; SD, Standard Deviation; PHQ-9, Patient Health Questionnaire-9; GAD-7, Generalized Anxiety Disorder-7; HSCL-11, Hopkins Symptom Checklist-11.

* Baseline measures of PHQ-9 and GAD-7 were assessed at the first session.

### Quantitative results

Results of all the models conducted are presented in Table 4.

**Table 4:** Results from the unconditional model, the model with time as the only predictor and the model of the effect of depression and anxiety on severity levels.

Effects models	HSCL-11 level		HSCL-11 rate of change during treatment	
	γ	*SE*	γ	*SE*
**Two-level unconditional model**				
Intercept	0.81^*^	0.09		
**Time as the only predictor (Level 1)**				
FME Intercept	0.62^*^	0.09	−0.09^*^	0.02
Model comparison	χ^2^(1) = 28.99, *p* < 0.001			
**Two-level conditional model**				
FME Intercept	0.03	0.14	−0.03	0.04
FME—PHQ-9 level (between-patient)	0.74^*^	0.15	−0.03	0.04
FME—GAD-7 level (between-patient)	−0.08	0.12	−0.02	0.03
Model comparison	χ^2^(4) = 46.87, *p* < 0.001			

Both the Model with Time as the Only Predictor and the Conditional Models were with the Two-Level Unconditional Model. PHQ-9, Patient Health Questionnaire-9; GAD-7, Generalized Anxiety Disorder-7; HSCL-11, Hopkins Symptom Checklist-11; FME, Fixed Model Effects.

* *p* < 0.001.

### Unconditional model

The model estimated a symptomatologic severity mean of 0.81 during the first five treatment sessions, γ_00_ = 0.81, SE = 0.09, 95% CI [0.64, 0.98], *t*(38) = 9.32, *p* < 0.001. Results showed that in a two-level unconditional model 75% of the variance in severity was explained by the differences between patients (ICC = 0.75), suggesting the appropriateness of conducting HLMs (Gómez Penedo et al., 2019).

### Time as only predictor model

We ran a fixed model which showed a significant change in clinical severity during the first five sessions, γ_10_ = −0,09, SE = 0.02, 95% CI[−0.12, −0.05], *t*(132) = −5.60, *p* < 0.001. Results show that symptom severity decreased by on average 0.09 units session-by-session. As an effect size measure, the standardized coefficient suggests that patients reduced their clinical severity 0.15 standard deviations every session. Including time as a linear predictor, improved the model fit compared with the unconditional one, χ^2^(1) = 28.99, *p* < 0.001. When including time as a random effect, there was no improvement in model fit, χ^2^(2) = 2.43, *p* = 0.30. Furthermore, the quadratic model did not improve the model fit when compared with the linear model, χ^2^(1) = 0.46, *p* = 0.50. Therefore, we selected the fixed linear model as the final model that best described the evolution of the clinical severity over time.

### Conditional model

Based on the results of the models with time as the only predictor, a two-level conditional model was fitted with initial depression and anxiety as level 2 predictors of both the intercept and the slope. The latter model represented a significant increase in model fit compared to the time-as-only predictor model: χ^2^(4) = 46.87, *p* < 0.001.

Results showed no significant effect of patients’ baseline anxiety and depression levels on clinical severity change session-by-session. However, there was a significant effect of baseline depression on the estimated severity at the end of the fifth session (γ_02_ = 0.74, SE = 0.15, 95% [0.46, 1.03], *t*(84) = 5.09, *p* < 0.001). Every one-unit greater depression severity at baseline was associated with 0.74 units greater clinical severity at the end of fifth session. Standardized coefficients as an effect size measure, showed that a one standard deviation greater baseline depression severity was related with a 0.71 standard deviations greater clinical severity at the end of the fifth session.

### Qualitative results

Based on the qualitative analyses, the material of the feedback provided by the therapists was organized into four thematic *domains*: (1) Perception of the information provided, (2) Perspectives on sharing assessed information with the patient, (3) Match between information assessed information and clinical judgment of the therapist, and (4) Suggestions about the feedback system design.

#### Perception of the information provided

In this domain, therapists expressed how they felt about the information given through the feedback device. Answers were classified into four main categories. The first category included all the therapists of the sample (*n* = 13) who reported that they found the given information useful and easy to understand. In the second category, five of the therapists stated that the information helped to guide their clinical practice. As an example, one of the therapists expressed *“(Based on this information) I could get a more precise diagnosis for the patient.”* The third category included four cases where therapists perceived that the graphic information was not completely clear. Particularly, they expressed that it was difficult to interpret the baseline graphic. Moreover, the last category included qualitative material from three therapists that reported that the information allowed them to better know about the patients’ clinical evolution.

#### Perspectives on sharing assessed information with the patient

In this domain, we found two main categories. The first one with 11 answers was “Therapist would share the received information with their patients” (*n* = 11) expressing they thought this would be useful also for patients. The second one with five answers established “*Sharing the feedback information could have a positive impact on the therapeutic process*.” Particularly expressing it would give the patient factual information and help, in case it is needed, to redirect the process.

#### Match between assessed information and clinical judgment of the therapist

In this domain, we developed three categories that link whether feedback information and clinical judgment concur or not. Eight therapists answered that generally baseline and evolution levels matched therapists’ perception. Seven therapists answered that sometimes both criteria did not come together. Finally, three therapists answered that this discrepancy turned out to be useful, specifically expressing that: “*It gave the opportunity to propose other strategies or also to enhance the interpretation of the case*.”

#### Suggestions about the feedback system design

This last domain included suggestions that the therapists presented for the feedback system. Four therapists affirmed that it will be important to have some guidelines to interpret the feedback. For example: *“It would be useful to have a guideline to interpret feedback and integrate it into treatment. Also for communicating it to the patient.”* This indicated a need for training in this practice of receiving and giving feedback. Moreover, three therapists expressed the need for simpler figures to present the results. For example, “*Simpler graphics or with more specific information would help the therapist with the interpretation*.”

## Discussion

The goal of the present pilot study was to present the results of an outcome monitoring and feedback system implemented in Argentina. Our main findings can be divided into quantitative and qualitative results.

Our quantitative results showed a significant reduction in patients’ severity (HSCL-11) during the first five sessions of treatment. This finding suggests the sensitivity of the measure to evaluate change. The HSCL-11 was able to assess the change trajectory during treatment, which is useful to provide feedback on patients’ evolution. The rate of change in the first four or five sessions is denominated *early response* and has been extensively studied in recent years (e.g., Lutz et al., 2014; Nordberg et al., 2014; Kyron et al., 2019). Early responses constitute well-established robust predictors of psychotherapy outcome (Beard and Delgadillo, 2019). The results of the present study suggest that implementing ROM and feedback in the first sessions of therapy could provide relevant information for the clinicians regarding early treatment responses, which in turn would impact the likelihood of achieving better psychotherapy outcomes.

The statistical analysis showed also that baseline depressive severity was a significant predictor of patients’ overall severity at the end of the fifth session, while anxiety severity was not a significant predictor. Considering that most of the patients with a diagnosis provided by the clinicians had an anxiety disorder (20 out of 34; 59%), these patients are expected to be more homogenous on their anxiety baseline severity (with rather high levels of anxiety symptoms), while presenting more variability on their depression severity (i.e., some patients having more depressive symptoms than others). That variability plus the fact that depressive severity in the context of anxiety disorders might be a proxy of complexity of the cases in terms of comorbid symptomatology might explain why depressive symptoms severity at baseline were a better predictor of early outcome in HSCL-11.

**Figure 3:**
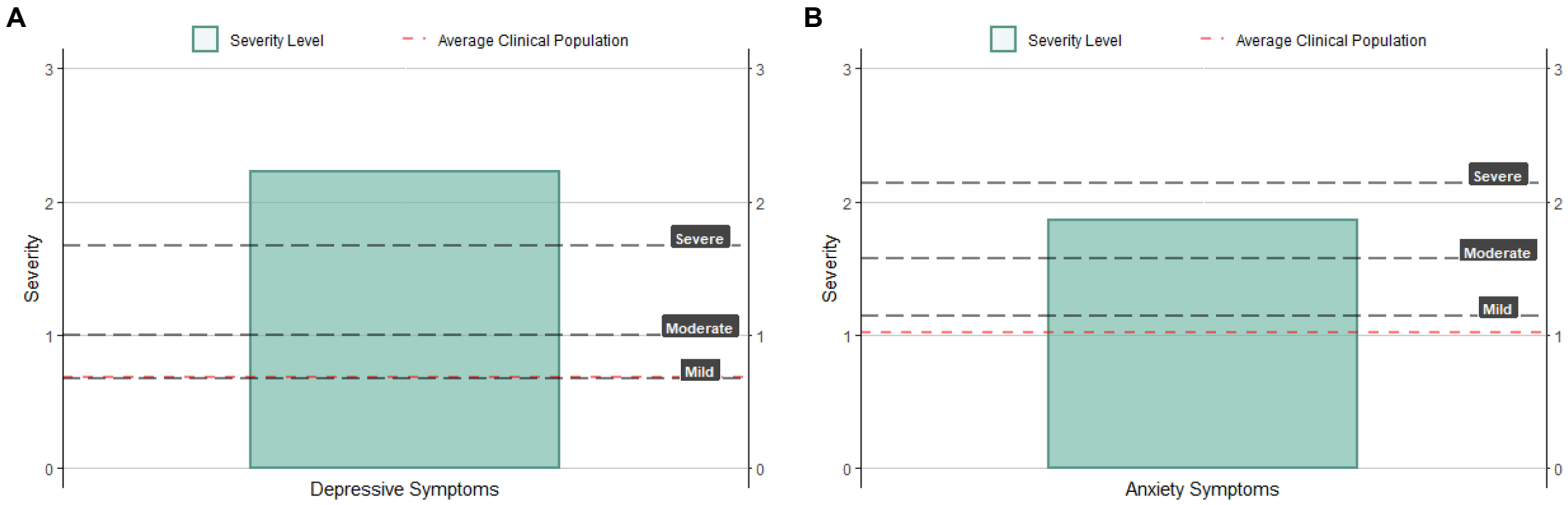
Examples of the revised plots developed to provide feedback to the therapists about patients baseline depression **(A)** and anxiety levels **(B)**.

The qualitative analysis showed that the ROM and feedback system were perceived as useful by therapists, and they stated that it helped them in their clinical practice. Therapists also mentioned that their clinical assessment was largely consistent with the feedback provided and that they would be willing to share that information with their patients. These results are aligned with previous findings presented by other qualitative studies in which therapists valued feedback of their patients and their respective progress (e.g., Solstad et al., 2021). This allows them to have an outside perspective besides inputs from supervisors. However, therapist also mentioned that it would be important to provide clearer guidelines for the implementation of the feedback system. This information could help therapists to be more engaged in the feedback system which could, in turn, have positive consequences on their patients (Moltu et al., 2018). The positive reaction to the feedback system could be explained by its user-friendly implementation, the short time needed by therapists to complete it, and the scarce availability of other feedback systems in the region. In addition, the system was not performed by an institution or psychotherapy center, which may have helped to lessen the feeling of an institutional audit (Wolpert, 2014). The suggestions provided by the therapists, especially the ones focused on the need of providing some guidelines and to enhance interpretability of the figures, were then incorporated when creating a new, self-explainable, version of the feedback for the therapists. Figure 3A shows an example of the new graphical visualizations developed based on therapists’ opinions to provide feedback of baseline PHQ-9. In this case, the patient showed a high level of depression symptomatology. Figure 3B shows an example of the graphical visualization of levels on the GAD-7 baseline measure of a patient with high levels of anxiety. The graphic includes patient’s severity measure, clinical population average and severity level lines.

**Figure 4:**
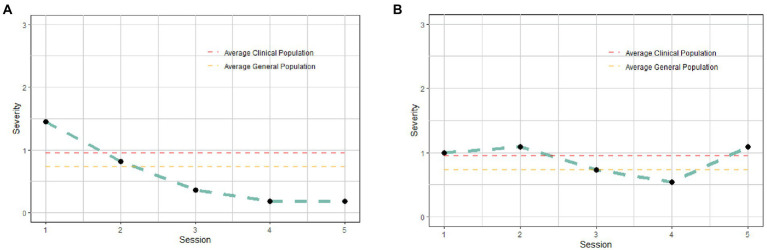
Examples of the revised plots provided to the therapists to give feedback about patients’ clinical evolution during the first five sessions. **(A)** presents an example of a patient who improve during the timeframe, while **(B)** presents the example of a patient who presented an stable pattern.

Furthermore, we also tried to enhance interpretability of the plots to provide feedback on patients’ clinical evolution. To the original plots (i.e., Figure 2), we incorporated references to average clinical and general population means (see Figure 4).

Despite the promising findings, this study should be considered in light of several limitations. First, as a pilot study the study does not have a control group and the sample may have been underpowered for the multilevel analysis. Future research would need to replicate these findings with a fully-powered design, grounded on the preliminary data from this pilot study. Besides, the study lacks diversity both in patients’ diagnoses and therapists’ theoretical orientations, limiting the generalization of the results. At the same time, future implementation studies of this ROM and feedback system for particular disorders and theoretical frameworks might provide relevant information about possible adaptations of the system to adjust for disorder and framework-specific needs. Additionally, as the main outcome measure, we used a brief symptomatic severity scale. Although symptoms severity is one of the main outcomes of psychotherapy there are other relevant dimensions that would be important to consider. Future ROM and feedback systems might benefit from using brief but more comprehensive measures of outcome that would incorporate other relevant dimensions such us patient’s functionality and interpersonal relations. Moreover, the treatments were conducted on-line, leaving the question regarding how this system would work in in-person psychotherapy unanswered. Furthermore, the study did not assess the patients’ perspective on the ROM and feedback system. We focused our study on the perspective of the therapists because they constitute the main target of a feedback system. However, incorporating the patients’ perspective might also provide relevant insights to improve both the ROM and feedback system and contribute with important information for the therapists when implementing the system. Finally, the qualitative data collection was structured, which implies that certain topics may not have arisen in therapist’s spontaneous answers, and we may have missed other important information.

Clinically the results of the present study point to a promising ROM and feedback system, which is a much-needed endeavor in the region. This system could be used as a clinical tool in different psychotherapy centers across Argentina, especially considering its low-cost implementation and its benefits. In this regard, a recent study that implemented ROM and feedback in a large university outpatient clinic explored the potentially interactive effect of the attitude and confidence of therapists using the system. The adherence to these systems determined their effectiveness (Lutz et al., 2022), which is in line with the fact that supplementing the use of ROM and feedback systems with training for therapists, increments their usefulness (de Jong et al., 2021).

Future research should be oriented to address the limitations previously mentioned to replicate this feedback system in different psychotherapy treatment settings and in bigger samples. It is also important to incorporate the patients’ perspectives to gain knowledge of their thoughts and experiences using the systems.

In conclusion, we present a promising clinical feedback tool with preliminary evidence showing its implementation potential. This constitutes a very important step forward in the pursuit of assessing therapeutic change and develop predictions about patients’ trajectories within a local system in Latin America. Considering the literature suggesting that ROM and feedback have a positive impact in several domains (de Jong et al., 2021), a validated system with these features is of utmost relevance for the practice of psychotherapy in Argentina.

## Data availability statement

The datasets presented in this article are not readily available because participants did not give consent to share data. Requests to access the datasets should be directed to jmgomezpenedo@gmail.com.

## Ethics statement

The studies involving human participants were reviewed and approved by Comité de Conductas Responsables en Investigación, Secretaría de Investigaciones, Universidad de Buenos Aires. The patients/participants provided their written informed consent to participate in this study.

## Author contributions

JG-P has participated in the study design, data analysis, and led the writing process. RMa has worked on the data collection, communication with the therapists, creating the initial feedback system, and the preliminary data analysis. MA and JF-Á have contributed with the data collection and the literature search for the study. MM has contributed with the coding for data analysis and the development of the revised feedback system for the therapists. AB had contributed with the design of the study and coordinating the work of the JG-P, RMa, MA, JF-Á, MM, SJ, ARon, RMu, ARou, WL, and MH. SJ and ARon have contributed with the qualitative analysis of the study. Finally, as senior researchers, RMu, ARou, WL, and MH have contributed to the design of the project and supervised the whole process of data collection, analysis, and result interpretation. All authors contributed to the article and approved the submitted version.

## Funding

This study was funding by a Seed grant from the Leading House for the Latin American Region (PI: JG-P and MG).

## Conflict of interest

The authors declare that the research was conducted in the absence of any commercial or financial relationships that could be construed as a potential conflict of interest.

## Publisher’s note

All claims expressed in this article are solely those of the authors and do not necessarily represent those of their affiliated organizations, or those of the publisher, the editors and the reviewers. Any product that may be evaluated in this article, or claim that may be made by its manufacturer, is not guaranteed or endorsed by the publisher.
